# *CHRNA7* Polymorphisms and Response to Cholinesterase Inhibitors in Alzheimer's Disease

**DOI:** 10.1371/journal.pone.0084059

**Published:** 2013-12-31

**Authors:** Pei-Hsuan Weng, Jen-Hau Chen, Ta-Fu Chen, Yu Sun, Li-Li Wen, Ping-Keung Yip, Yi-Min Chu, Yen-Ching Chen

**Affiliations:** 1 Department of Family Medicine, Taiwan Adventist Hospital, Taipei, Taiwan; 2 Institute of Epidemiology and Preventive Medicine, College of Public Health, National Taiwan University, Taipei, Taiwan; 3 Department of Geriatrics and Gerontology, National Taiwan University Hospital, Taipei, Taiwan; 4 Department of Neurology, National Taiwan University Hospital, Taipei, Taiwan; 5 Department of Neurology, En Chu Kong Hospital, Taipei, Taiwan; 6 Department of Laboratory Medicine, En Chu Kong Hospital, Taipei, Taiwan; 7 Center of Neurological Medicine, Cardinal Tien Hospital, Taipei, Taiwan; 8 School of Medicine, Fu-Jen Catholic University, Taipei, Taiwan; 9 Department of Laboratory Medicine, Cardinal Tien Hospital, Taipei, Taiwan; 10 Department of Public Health, College of Public Health, National Taiwan University, Taipei, Taiwan; 11 Research Center for Genes, Environment and Human Health, College of Public Health, National Taiwan University, Taipei, Taiwan; Kyushu University, Japan

## Abstract

**Background:**

*CHRNA7* encodes the α7 nicotinic acetylcholine receptor subunit, which is important to Alzheimer's disease (AD) pathogenesis and cholinergic neurotransmission. Previously, *CHRNA7* polymorphisms have not been related to cholinesterase inhibitors (ChEI) response.

**Methods:**

Mild to moderate AD patients received ChEIs were recruited from the neurology clinics of three teaching hospitals from 2007 to 2010 (n = 204). Nine haplotype-tagging single nucleotide polymorphisms of *CHRNA7* were genotyped. Cognitive responders were those showing improvement in the Mini-Mental State Examination score ≧2 between baseline and 6 months after ChEI treatment.

**Results:**

AD women carrying rs8024987 variants [GG+GC vs. CC: adjusted odds ratio (AOR) = 3.62, 95% confidence interval (CI) = 1.47–8.89] and GG haplotype in block1 (AOR = 3.34, 95% CI = 1.38–8.06) had significantly better response to ChEIs (false discovery rate <0.05). These variant carriers using galantamine were 11 times more likely to be responders than female non-carriers using donepezil or rivastigmine**.**

**Conclusion:**

For the first time, this study found a significant association between *CHRNA7* polymorphisms and better ChEI response. If confirmed by further studies, *CHRNA7* polymorphisms may aid in predicting ChEI response and refining treatment choice.

## Introduction

Loss of cholinergic neurons and nicotinic acetylcholine receptors (nAChR) is one of the pathological hallmarks of Alzheimer's disease (AD) [1], [2]. Cholinesterase inhibitors (ChEIs), the most commonly prescribed medication for mild to moderate AD, were designed to increase cholinergic neurotransmission [3]. However, ChEIs are costly and produce only moderate effects, while strong and consistent predictors of response are lacking.

Genetic variations may account for a major proportion of the individual differences in drug efficacy. Genetic polymorphisms of apolipoprotein E (*APOE*), butyrylcholinesterase, paraoxonase-1, choline acetyltransferase and acetylcholinesterase have been related to different ChEI treatment response but with inconsistent findings [4]–[11]. Cytochrome 2D6 (*CYP2D6*) polymorphisms have been shown to affect the metabolism and treatment response of ChEIs [10], [12], [13]. However, the application of *CYP2D6* genetic markers is limited because of the complexity of ChEI metabolism [14] and the effect of concomitant medications in the elderly. Recently, two single nucleotide polymorphisms (SNPs) in *PRKCE* and *NBEA* genes were associated with ChEI response in a genome-wide association study (GWAS) [15]. However, one pitfall of the standard GWAS approach is that it might fail to identify important genetic variants under gene-environment interactions, when the genetic association is opposite among different subgroups [16]. Therefore, it is important to identify new genetic markers to predict ChEI response.

nAChRs are ligand-gated ion channels that mediate the effect of the neurotransmitter acetylcholine. α7 nAChR, encoded by *CHRNA7* on chromosome 15q14, is one of the major nAChR subunits in the central nervous system. Recent studies have shown that *CHRNA7* polymorphisms were associated with reduced AD risk and slower progression from mild cognitive impairment to AD [17], [18]. In addition, α7 nAChR plays an important role in the cholinergic neurotransmission relating to ChEI action [19]. Among ChEIs, galantamine was also known to have a unique positive allosteric modulation effect on α7 nAChR [20], [21], which potentiates the effect of acetylcholine. However, no studies have assessed the association between *CHRNA7* polymorphisms and cognitive response to ChEI treatment. This study aimed to investigate this association and whether the association varies by ChEI types. Stratified analyses were performed by gender because of the sex dimorphisms in the expression of cholinergic systems [22]–[25], which may alter the effects of *CHRNA7* polymorphisms on ChEI response.

## Materials and Methods

### Ethics Statement

The study protocol was approved by the Institutional Review Boards of the three hospitals (National Taiwan University Hospital, En Chu Kong Hospital, Cardinal Tien Hospital). Written consents from legal guardian/next of kin were obtained when the patients had severe cognitive impairment.

### Study Population

This is a retrospective cohort study. A total of 223 mild to moderate AD patients, aged 65 years or older and receiving ChEI treatment, were recruited from the neurology clinics of three teaching hospitals in northern Taiwan from November 2007 to July 2010. All of the participants were Taiwanese. The study design was detailed elsewhere [26]. Participants who took memantine before or at the time of entering this study were excluded (n = 2). After the initiation of ChEI treatment, 17 patients were further excluded for the following reasons: loss to follow-up (n = 11), discontinuation of ChEIs due to side effects (n = 1), poor drug compliance (n = 1), lack of Mini-Mental State Examination (MMSE) data at the 6^th^ month (n = 1), and poor DNA quality (n = 3). After exclusion of these patients, a total of 204 patients who were 6-month ChEI completers were included in the statistical analyses.

A detailed questionnaire was administered to all the participants via a face-to-face interview with the assistance of their family members. The collected information included data on demography, lifestyle, comorbidity, and family history of dementia. Blood samples were collected in EDTA tubes, and genomic DNA was extracted from buffy coat by QuickGene-Mini80 kit (Fujifilm, Tokyo, Japan) after centrifugation.

### Evaluation of AD and Cognitive Response to ChEI Treatment

Probable AD was diagnosed by experienced neurologists according to the National Institute of Neurological and Communicative Disorders and Stroke and the Alzheimer's Disease and Related Disorders Association criteria [27]. A designation of mild to moderate AD was given to patients with MMSE scores 10 to 26 [28]. Brain images (computed tomography or magnetic resonance imaging) were performed to exclude organic brain lesions.

The types of prescribed ChEIs were selected by the neurologists based on patients' clinical conditions. A Chinese version of MMSE [28], [29] was performed at baseline and six months after the initiation of ChEIs according to the guidelines of Taiwan's National Health Insurance Bureau. Data on medication history, adverse drug reactions, and MMSE scores was collected over time via chart review. The prescribing physicians and the personnel who performed the neuropsychological tests were blinded to the genotyping results.

The outcome of this study was cognitive response to ChEIs. Cognitive responders to ChEIs were defined as those showing an improvement in MMSE score ≥two points between baseline and 6 months after ChEI treatment. The two-point MMSE threshold corresponded to a four-point decline in the Alzheimer's Disease Assessment Scale-Cognition Subscale, which was considered a clinically significant response by the U.S. Food and Drug Administration [30], [31].

### SNP Selection and Genotyping Assays

Common (frequency ≥5%) SNPs in *CHRNA7* were selected from Han Chinese in Beijing (CHB) genotype data from the International HapMap Project (http://hapmap.ncbi.nlm.nih.gov/). Haplotype blocks were determined by the Haploview program (http://www.broadinstitute.org/haploview/haploview) using a modified Gabriel algorithm [32], [33]. Haplotype-tagging SNPs (htSNPs) were selected from each haplotype block using the tagSNP program with R^2^>0.7 in each haplotype block [34].

Genotypes of *CHRNA7* SNPs were determined by TaqMan® Genomic Assays using the ABI 7900HT fast real-time PCR system (Applied Biosystems Inc., CA, USA). *APOE* genotypes were determined by the assay developed by Chapman et al [35]. The genotyping call rate was greater than 95% for each SNP. The internal genotyping quality control obtained from 5% of samples in duplicates had a concordance rate of 100%.

### Statistical Analyses

The Hardy-Weinberg equilibrium (HWE) test was performed for each SNP of *CHRNA7* and *APOE* genes by response status. The expectation-maximization algorithm was applied to estimate haplotype frequencies [34]. Logistic regression models were used to estimate adjusted odds ratio (AOR) and 95% confidence intervals (CIs) for ChEI cognitive response in participants carrying 1 or 2 versus 0 copies of the minor allele of each SNP and each multilocus haplotype. Variables adjusted in the logistic regression models, e.g., age, baseline MMSE, *APOE* ε4 status, and hypertension history, were selected either by stepwise selection or because they were found to be important determinants of ChEI response in previous studies [36]-[38]. Type of ChEIs was not a significant determinant of treatment response, so this covariate was not adjusted in the final model. Stratified analyses were performed by gender because of the sex dimorphisms mentioned above [22]–[25]. Correction for multiple tests was performed by false discovery rate (FDR) using method of Benjamini and Hochberg (1995) [39].

Different ChEIs work through different mechanisms via α7nAChR. Therefore, we further explored whether patients carrying certain genotypes had better treatment response to specific types of ChEIs. Donepezil, rivastigmine, and galantamine were the prescribed ChEIs. Galantamine has a unique dual mode of biological action, i.e., inhibition of acetylcholinesterase and allosteric modulation of nAChR [20]. In addition, the genetic effects on cognitive response were similar among donepezil and rivastigmine users in this study. Therefore, donepezil and rivastigmine were pooled together as non-galantamine ChEIs. To compare the effect of *CHRNA7* polymorphisms on cognitive response to different types of ChEIs, four categories were created for each SNP (non-variant carriers of *CHRNA7* SNP using non-galantamine ChEIs/galantamine, variant carriers using non-galantamine ChEIs/galantamine). Participants who were non-variant carriers and used non-galantamine ChEIs served as the reference group.

Sensitivity analyses were further performed using the intention-to-treat approach, in which those who dropped out before 6 months of ChEI treatment were included and analyzed as non-responders. For analyses with small sample sizes after stratification, exact logistic regression was repeated to ensure the robustness of the results [40]. A co-dominant model was utilized to assess the mode of inheritance (data not shown). Because most of the SNPs followed dominant mode of inheritance, all analyses were carried out under dominant model. All statistical analyses were performed using SAS 9.2 (SAS Institute, Cary, NC, USA). A two-sided *P*-value of less than 0.05 was considered statistically significant.

## Results

### Characteristics of Study Population

This study included 204 AD patients who received ChEI treatment, and 63% of whom were women (n = 128). About 30% of participants (n = 61) were classified as cognitive responders. The mean baseline MMSE score (18.3) was significantly lower among cognitive responders than among non-responders (20.3, *P*<0.01). The distributions of the following variables were similar between ChEI non-responders and responders: age, sex, education level, cigarette smoking, alcohol consumption, vascular risk factors (hypertension and type 2 diabetes mellitus), *APOE* ε4 status, types of ChEIs, and drug-related side effects (Table 1).

**Table 1:** Baseline characteristics and ChEI types of the study population.

	Non-responder (n = 143)	Responder (n = 61)
	mean ± SD	
Age	78.4±5.6	77.7±5.5
Baseline MMSE	20.3±4.0	18.3±4.5*
	n (%)	
Female	93(65)	35 (57)
Education		
≦6 years	72 (51)	32 (53)
>6 years	70 (49)	28 (47)
Cigarette smoking	32 (23)	8(14)
Alcohol consumption	12 (8)	5 (8)
Hypertension	50 (35)	26 (43)
Type 2 diabetes mellitus	35 (24)	10 (16)
*APOE* ε4 status	57 (40)	21 (34)
ChEI type		
Donepezil		
5 mg/day	81 (56)	37 (61)
10 mg/day	1 (1)	2 (3)
Rivastigmine		
4.5 mg/day	2 (1)	0 (0)
6 mg/day	7 (5)	1 (2)
9 mg/day	27 (19)	7 (11)
Galantamine		
8 mg/day	1 (1)	0 (0)
16 mg/day	24 (17)	14 (23)
ChEI side effect	11 (8)	4 (7)

Abbreviations: SD, standard deviation; MMSE, Mini-Mental State Examination; *APOE*, apolipoprotein E; ChEI, cholinesterase inhibitor.

*P*<0.01 for comparing responders to non-responders.

### Haplotype-tagging SNPs in *CHRNA7*

**Figure 1:**
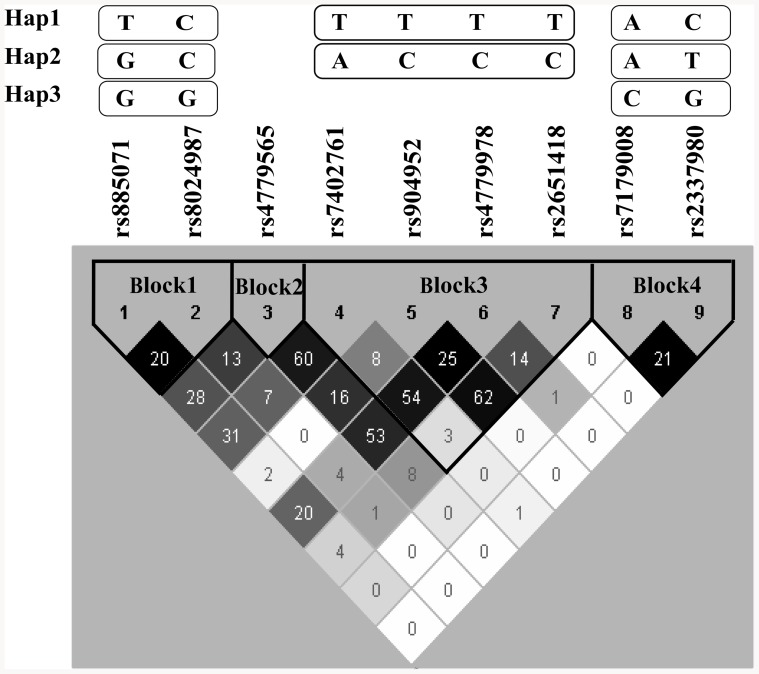
*CHRNA7* linkage disequilibrium (LD) plot. The plot was generated by applying the Haploview program to genotype data from this study. The level of pairwise D', which indicates the degree of LD between two SNPs, is shown in the LD structure in gray scale. The level of pairwise r^2^, which indicates the degree of correlation between two SNPs, is indicated by the number in the cell. Different numbers of common (frequency ≥5%) haplotypes were identified in each haplotype block. A modified Gabriel et al. algorithm was used to define the haplotype block [32], [33].

Nine common (frequency ≥5%) htSNPs forming four haplotype blocks of the *CHRNA7* gene were selected and genotyped (Figure 1, Table 2). Block1 contained two htSNPs (SNP1: rs885071; SNP2: rs8024987), block2 contained one htSNP (SNP3: rs4779565), block3 contained 4 htSNPs (SNP4: rs7402761; SNP5: rs904952; SNP6: rs4779978; SNP7: rs2651418), and block4 contained 2 htSNPs (SNP8: rs7179008; SNP9: rs2337980). The linkage disequilibrium (LD) structure was shown in Figure 1. In this study, the minor allele frequencies (MAFs) of the nine htSNPs ranged from 0.05 to 0.48. None of the *CHRNA7* SNPs were out of HWE in either responders or non-responders.

**Table 2:** Characteristics of *CHRNA7* haplotype-tagging SNPs.

Haplotype	SNP	rs no.	Nucleotide	Location	Non-responder		Responder	
block	name		change		MAF	HWE *p*	MAF	HWE *p*
1	SNP1	rs885071	T→G	Intron	0.45	0.88	0.48	0.26
1	SNP2	rs8024987	C→G	Intron	0.13	0.25	0.20	0.65
2	SNP3	rs4779565	G→T	Intron	0.39	0.61	0.40	0.54
3	SNP4	rs7402761	A→T	Intron	0.44	0.99	0.46	0.94
3	SNP5	rs904952	T→C	Intron	0.33	0.31	0.34	0.20
3	SNP6	rs4779978	C→T	Intron	0.36	0.18	0.34	0.90
3	SNP7	rs2651418	T→C	Intron	0.43	0.67	0.43	0.32
4	SNP8	rs7179008	A→G	Intron	0.07	0.10	0.05	0.68
4	SNP9	rs2337980	C→T	Intron	0.23	0.29	0.27	0.73

Abbreviations: SNP, single nucleotide polymorphism; MAF, minor allele frequency; HWE, Hardy–Weinberg equilibrium test.

### *CHRNA7* SNPs and Cognitive Response to ChEI Treatment

Compared with non-variant carriers, women carrying one or two copies of variant SNP2 had a better cognitive response to ChEI treatment (GG+GC vs. CC: AOR = 3.62, 95% CI = 1.47–8.89, *P* = 0.005, Table 3). This association remained significant after controlling for FDR. However, no significant association was observed among men for any of the nine SNPs. A significant interaction was found between gender and SNP2 (*P_interaction_* = 0.01, Table 3).

**Table 3:** Association between *CHRNA7* SNPs, haplotypes, and ChEI response by gender.

		Dominant model				
Haplotype	SNP/					
block	Haplotype	0 copies		1 or 2 copies		
	(frequency among	Responder/	AOR	Responder/	AOR (95% CI)	*P*_interaction_
	all ChEI users)	Non-responder		Non-responder		
1	SNP1 All	19/43	1.00	42/100	1.08 (0.55–2.15)	0.43
	F	10/29	1.00	27/64	1.32 (0.53–3.27)	
	M	9/14	1.00	15/36	0.76 (0.26–2.24)	
1	SNP2 All	38/106	1.00	21/33	1.70 (0.85–3.39)	0.01
	F	19/74	1.00	16/18	3.62 (1.47–8.89)*	
	M	19/32	1.00	5/15	0.55 (0.16–1.82)	
2	SNP3 All	23/51	1.00	38/90	0.92 (0.48–1.77)	0.29
	F	11/31	1.00	26/62	1.22 (0.51–2.94)	
	M	12/20	1.00	12/28	0.62 (0.22–1.74)	
3	SNP4 All	18/44	1.00	43/98	1.14 (0.57–2.26)	0.62
	F	10/27	1.00	27/66	1.28 (0.51–3.21)	
	M	8/17	1.00	16/32	1.02 (0.34–3.01)	
3	SNP5 All	28/66	1.00	31/75	1.08 (0.56–2.07)	0.45
	F	17/41	1.00	18/52	0.91 (0.39–2.15)	
	M	11/25	1.00	13/23	1.41 (0.51–3.93)	
3	SNP6 All	26/62	1.00	35/80	1.04 (0.54–1.99)	0.62
	F	15/39	1.00	22/54	1.15 (0.49–2.68)	
	M	11/23	1.00	13/26	0.98 (0.33–2.85)	
3	SNP7 All	22/48	1.00	39/94	0.97 (0.50–1.88)	0.15
	F	15/27	1.00	22/66	0.68 (0.29–1.61)	
	M	7/21	1.00	17/28	1.85 (0.62–5.51)	
4	SNP8 All	53/124	1.00	6/18	0.83 (0.30–2.30)	0.85
	F	30/79	1.00	5/14	0.84 (0.26–2.69)	
	M	23/45	1.00	1/4	0.69 (0.07–6.92)	
4	SNP9 All	33/83	1.00	28/59	1.38 (0.72–2.63)	0.85
	F	18/51	1.00	19/42	1.42 (0.62–3.26)	
	M	15/32	1.00	9/17	1.22 (0.42–3.54)	
1	Hap1: TC All	16/30	1.00	45/113	0.91 (0.44–1.90)	0.30
	(54%) F	10/16	1.00	27/77	0.68 (0.26–1.77)	
	M	6/14	1.00	18/36	1.46 (0.45–4.78)	
1	Hap2: GC All	33/64	1.00	28/79	0.72 (0.38–1.36)	0.46
	(31%) F	21/41	1.00	16/52	0.56 (0.25–1.29)	
	M	12/23	1.00	12/27	0.95 (0.34–2.64)	
1	Hap3: GG All	40/110	1.00	21/33	1.67 (0.84–3.30)	0.01
	(15%) F	21/75	1.00	16/18	3.34 (1.38–8.06)*	
	M	19/35	1.00	5/15	0.54 (0.16–1.79)	

Abbreviations: SNP, single nucleotide polymorphism; ChEI, cholinesterase inhibitor;

M, male; F, female; AOR, adjusted odds ratio; CI, confidence interval.

All models were adjusted for age, baseline MMSE, hypertension, and *APOE* ε4 status.

The association remained significant after correction for multiple tests by false discovery rate (FDR) among AD women.

### *CHRNA7* Haplotypes and ChEI Cognitive Response

Three common haplotypes were identified in haplotype block1. Block2 included only one htSNP and was thus excluded from the haplotype analysis. Three out of five common haplotypes in block3 were singletons and were thus excluded. Block4 included three common haplotypes. AD women carrying Hap3 GG of block1 were more likely to be ChEI responders compared with non-carriers (AOR = 3.34, 95% CI = 1.38–8.06, *P* = 0.007, Table 3), which remained significant after controlling for FDR. No significant associations were observed for haplotypes in block3 or block4 (Table S1). A significant interaction was identified between gender and Hap3 in block1 (*P*_interaction_ = 0.01, Table 3).

### Effect Modification by Types of ChEI

In AD women, SNP2 variant carriers using galantamine were notably more likely to be cognitive responders (GG+GC vs. CC: AOR = 11.50, 95% CI = 1.89–69.93, Table 4) than non-carriers who took non-galantamine ChEIs. No significant associations were observed for other SNPs. A significant finding was observed for Hap3 in block1 (female galantamine users carrying 1 or 2 copies vs. 0 copies of Hap3: AOR = 10.14, 95% CI = 1.70–60.46). SNP2 or Hap3 in block1 did not significantly interact with ChEI types on the treatment response.

**Table 4:** *CHRNA7* SNP2 (rs8027987) and ChEI response by sex and ChEI type.

	SNP2				Block1 Hap3 (GG)	
	0 copies (CC)		1 or 2 copies (GG+GC)		0 copies	1 or 2 copies
	Responder/		Responder/			
	Non-responder	AOR (95% CI)	Non-responder	AOR (95% CI)	AOR (95% CI)	AOR (95% CI)
Female						
Non-galantamine	16/60	1.00	11/16	2.51 (0.91–6.96)	1.00	2.17 (0.80–5.87)
Galantamine	3/14	0.78 (0.18-3.44)	5/2	11.50 (1.89–69.93)^**^	0.71 (0.17–2.96)	10.14 (1.70–60.46)*
Male						
Non-galantamine	15/27	1.00	3/13	0.37 (0.09–1.58)	1.00	0.37 (0.09–1.61)
Galantamine	4/7	0.82 (0.18–3.74)	2/2	1.49 (0.17–13.22)	0.87 (0.19–3.93)	1.52 (0.17–13.53)

Abbreviations: SNP, single nucleotide polymorphism; ChEI, cholinesterase inhibitor; AOR, adjusted odds ratio; CI, confidence interval.

Non-galantamine refers to users of donepezil or rivastigmine.

*P*<0.05, ^**^ *P*<0.01.

All models were adjusted for age, baseline MMSE, hypertension, *and APOE* ε4 status.

All statistical analyses were replicated using the intention-to-treat approach and the results were similar (data not shown). For sparse cells in some strata (Table 4), exact logistic regression was repeated. The association between SNP2 variant carriers and response to galantamine remained significant in women, with a slightly reduced magnitude of the effect estimate (exact AOR = 8.51, 95% CI = 1.27–22.03, *P* = 0.02).

## Discussion

To the best of our knowledge, this study for the first time explored the association between *CHRNA7* polymorphisms and cognitive response to ChEI treatment. We found that variant *CHRNA7* rs8024987 (SNP2) and Hap3 GG in block1 were significantly associated with a better 6-month ChEI cognitive response in AD women, especially among galantamine users. This association was significantly modified by gender. This study showed that *CHRNA7* polymorphisms may be important predictors of ChEI response in treating AD patients, which bodes well for the future of personalized treatment.

SNP2 is an intronic SNP, which may affect the expression of α7 nAChR via pre-mRNA alternative splicing [41], [42] and the subsequent protein production [42]. It is also possible that the better response to ChEIs among SNP2 carriers was due to the LD between SNP2 and other functional SNPs that affect the cholinergic pathway. Among AD women, the response to ChEI treatment improved as the number of G alleles of SNP2 increased (co-dominant model: GC vs. CC: AOR = 3.40; GG vs. CC: AOR = 7.17; additive model: AOR = 3.53, 95% CI = 1.43–8.71, *P* for trend <0.01). SNP1 (rs885071) and SNP2 were the variant alleles in Hap3 GG of block1, which explains the protective effect of Hap3. Although SNP1 and SNP2 were in strong LD, the pairwise correlation between SNP1 and SNP2 was low (r^2^ = 0.20). This may illustrate the non-significant association of SNP1 and the ChEI cognitive response (Table 3).

**Figure 2:**
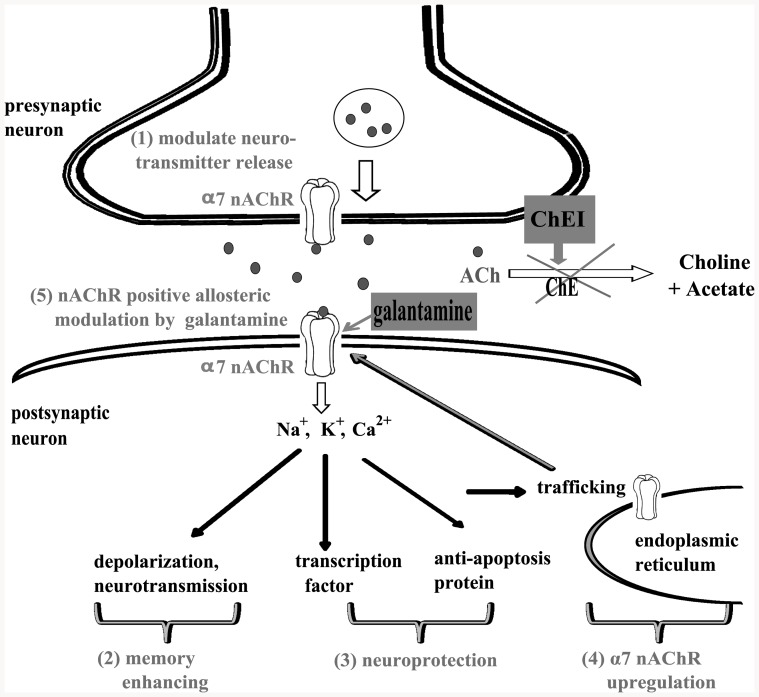
Postulated mechanism for *CHRNA7* polymorphisms and ChEI cognitive response. ChEIs increase the level of acetylcholine, which binds to α7 nAChR (encoded by *CHRNA7*). *CHRNA7* polymorphisms are postulated to affect the cognitive response to ChEIs through the following mechanisms: (1) modulation of neurotransmitter release in presynaptic neurons, (2) enhancement of memory via mediating cholinergic neurotransmission, (3) neuroprotection via α7 nAChR, (4) upregulation of α7 nAChR by ChEI, and (5) galantamine-associated positive allosteric modulation of α7 nAChR. Abbreviations: nAChR, nicotinic acetylcholine receptor; ACh, acetylcholine; ChE, cholinesterase; ChEI, cholinesterase inhibitor.

Our findings of a relationship between *CHRNA7* polymorphisms and ChEI cognitive response might be explained in several ways (Figure 2). First, ChEIs have a direct cognitive-enhancing effect through reducing the breakdown of acetylcholine, which binds to α7 nAChR and enhances cholinergic neurotransmission [19]. Some *in vitro* studies also showed that α7 nAChR is important in mediating the neuroprotective effect of ChEIs against the toxicity of amyloid β protein [43], [44]. α7 nAChR also modulates the release of neurotransmitters in presynaptic neurons [45]. In addition, chronic treatment with ChEIs further upregulates α7 nAChR, which induces a positive feedback loop and amplifies the effect of ChEIs [46]. Therefore, it is possible that *CHRNA7* polymorphisms improve ChEI cognitive response through the mechanisms above by modulating the expression of α7 nAChR.

In this study, AD women carrying the *CHRNA7* SNP2 variant had a markedly higher response rate to galantamine compared with other types of ChEIs. A distinct feature of galantamine is that its treatment effect derives more from the positive allosteric modulation of nAChR than its inhibition of cholinesterase [20], [47]. This allosteric modulation effect potentiates the neuronal responses to acetylcholine via α7 nAChR (Figure 2) [21]. Based on the mechanisms above, *CHRNA7* polymorphisms may be important pharmacogenetic markers for galantamine. However, due to the small number of galantamine users in the present study (n = 39, 19% of all ChEI users), our finding should be interpreted with caution and requires replication in large studies.

A significant interaction between gender and *CHRNA7* polymorphisms was observed. The effect of SNP2 variant was evident only among AD women but not among men. Sex differences have been reported in numerous central cholinergic markers previously, such as acetylcholine concentration [22], cholinesterase activity [25], and upregulation of nAChR [24]. However, past studies were limited to animals, and the underlying biological mechanisms in humans remain unclear. The opposite genetic effects among men and women might explain why *CHRNA7* polymorphisms were not identified in the previous pharmacogenetic GWAS [15], [16].

This study has several strengths. This is the first exploration of the association between genetic polymorphisms of *CHRNA7* and ChEI response in AD patients, which provides possible new directions for personalized treatment. In addition, SNPs selected in *CHRNA7* captured abundant genetic information (R^2^>0.7 in each haplotype block) and are representative for this Asian population.

This study also has some limitations. First, it is possible that the effect of SNP2 variants on ChEI response was attributable to a slower progression of cognitive decline rather than any modulation of the response to medication. However, our case-control studies on the same AD population revealed that SNP2 was not associated with reduced AD risk (unpublished data), which makes this possibility less likely. Second, this study included 76 AD men, which may not offer sufficient statistical power to assess the association in men. Also, the genetic effect on galantamine treatment response should be interpreted as merely hypothesis generating due to small number of galantamine users. Future large studies are necessary for hypothesis testing before considering galantamine as the treatment choice for AD women carrying SNP2 variants.

In summary, AD women carrying *CHRNA7* rs8024987 variant had a better 6-month cognitive response after ChEI treatment than non-carriers, especially among galantamine users. Polymorphisms of nAChR are promising candidates in AD pharmacogenetics. However, the biological functions of *CHRNA7* SNPs remain unclear, and how α7 nAChR polymorphisms affect its allosteric modulation on galantamine is also worth exploration. Although our findings offer the future possibilities of individualized treatment, large studies will be necessary to confirm the association and unravel the underlying mechanisms.

## Supporting Information

Table S1 **Association between** ***CHRNA7*** **haplotypes (block3, 4) and ChEI response by gender.**
(DOC)
