# Supplementary material for: CHRNA7 Polymorphisms and Response to Cholinesterase Inhibitors in Alzheimer's Disease
Source: PLoS One. 2013 Dec 31;8(12):e84059. doi: 10.1371/journal.pone.0084059 (PMC3877150; doi:10.1371/journal.pone.0084059)
Supplement: Table S1 — Association between CHRNA7 haplotypes (block3, 4) and ChEI response by gender. (DOC) [file pone.0084059.s001.doc]

|  |  | | **Dominant model** | | | | |
| --- | --- | --- | --- | --- | --- | --- | --- |
| **Haplo-** | **Haplotype** | | **0 copies** | | **1 or 2 copies** | |  |
| **type** | **(frequency among all ChEI** | | **Responder/** | **AOR** | **Responder/** | **AOR (95% CI)** | ***P*_interaction_** |
| **block** | **users)** | | **Non-responder** |  | **Non-responder** |  |  |
| 3 | Hap 1: TTTT | All | 34/80 | 1.00 | 27/63 | 1.02 (0.53-1.97) | 0.76 |
|  | (28%) | F | 20/52 | 1.00 | 17/41 | 1.12 (0.47-2.62) |  |
|  |  | M | 14/28 | 1.00 | 10/22 | 0.92 (0.31-2.79) |  |
| 3 | Hap 2 : ACCC | All | 56/133 | 1.00 | 5/10 | 0.85 (0.19-3.81) | 0.82 |
|  | (25%) | F | 34/87 | 1.00 | 3/6 | 1.01 (0.15-6.83) |  |
|  |  | M | 22/46 | 1.00 | 2/4 | 0.61 (0.05-7.42) |  |
| 4 | Hap 1: AC | All | 6/5 | 1.00 | 56/138 | 0.41 (0.10-1.62) | 0.78 |
|  | (76%) | F | 4/4 | 1.00 | 34/89 | 0.64 (0.12-3.36) |  |
|  |  | M | 2/1 | 1.00 | 22/49 | 0.10 (0.01-1.55) |  |
| 4 | Hap 2: AT | All | 37/125 | 1.00 | 24/18 | 1.69 (0.86-3.32) | 0.74 |
|  | (17%) | F | 22/79 | 1.00 | 15/14 | 1.65 (0.70-3.91) |  |
|  |  | M | 15/46 | 1.00 | 9/4 | 1.70 (0.57-5.08) |  |
| 4 | Hap 3: GT | All | 55/125 | 1.00 | 6/18 | 0.78 (0.28-2.17) | 0.74 |
|  | (6%) | F | 32/79 | 1.00 | 5/14 | 0.79 (0.25-2.50) |  |
|  |  | M | 23/46 | 1.00 | 1/4 | 0.68 (0.07-6.98) |  |

**Table S1**  Association between *CHRNA7* haplotypes (block3, 4) and ChEI response by gender

Abbreviations: M, male; F, female; AOR, adjusted odds ratio; CI, confidence interval; ChEI, cholinesterase inhibitor. The result of haplotype block 1 was shown in Table 3. Block 2 included only one haplotype-tagging single nucleotide polymorphism and thus was not included in the haplotype analysis.

All models were adjusted for age, baseline MMSE, hypertension, and *APOE* ε4 status.
